# Photocatalytic Degradation of Methylene Blue Using Polymeric Membranes Based on Cellulose Acetate Impregnated with ZnO Nanostructures

**DOI:** 10.3390/polym13193451

**Published:** 2021-10-08

**Authors:** Muna A. Abu-Dalo, Saja A. Al-Rosan, Borhan A. Albiss

**Affiliations:** 1 Department of Chemistry, Faculty of Science and Arts, Jordan University of Science and Technology, Irbid 22110, Jordan; 2 Nanomaterials Lab, Department of Physics, Faculty of Science and Arts, Jordan University of Science and Technology, Irbid 22110, Jordan; sajaalrousan93@gmail.com

**Keywords:** photocatalytic activity, polymeric membrane, methylene blue, ZnO nanostructures

## Abstract

This paper studied the photocatalytic degradation of methylene blue (MB) using polymeric membrane impregnated with ZnO nanostructures under UV-light and sunlight irradiation. ZnO nanoparticles and ZnO nanowires were prepared using the hydrothermal technique. Cellulose acetate polymeric membranes were fabricated by the phase inversion method using dimethylformamide (DMF) as a solvent and ZnO nanostructures. The structural properties of the nanostructures and the membranes were investigated using XRD, SEM, FTIR, and TGA measurements. The membranes were tested for photocatalytic degradation of MB using a UV lamp and a sunlight simulator. The photocatalytic results under sunlight irradiation in the presence of cellulose acetate impregnated with ZnO nanoparticles (CA-ZnO-NP) showed a more rapid degradation of MB (about 75%) compared to the results obtained under UV-light irradiation degradation (about 30%). The results show that CA-ZnO-NP possesses the photocatalytic ability to degrade MB efficiently at different levels under UV-light and sunlight irradiation. Modified membranes with ZnO nanoparticles and ZnO nanowires were found to be chemically stable, recyclable, and reproducible. The addition of ZnO nanostructure to the cellulose membranes generally enhanced their photocatalytic activity toward MB, making these potential membranes candidates for removing organic pollutants from aqueous solutions.

## 1. Introduction

Water is a valuable natural resource, and its quality and availability are essential for the survival of the earth’s living creatures. Industrial wastewater is one of the most significant sources of organic water pollutants during the last century due to excessive discharge into various natural water resources. In the last few years, parallel to the accelerated development of dye industries; this behavior has resulted in a serious water problem and caused negative effects on the environment and human lives. Methylene blue (MB) is a well known organic dye that forms a stable solution with water at room temperature [1]. MB is reported to be quite harmful when it exceeds specific concentrations, due to its high toxicity. Furthermore, most organic dyes do not biodegrade easily because they are highly resistant to environmental conditions, making their removal from wastewater both a critical need and a challenging task [2]. In this regard, it is essential to develop effective, low-cost, and novel materials for MB and other organic dye removal from aqueous solutions to bring life back to the environment.

Several methods have been used for removing dyes from aqueous solutions, including physical, chemical, and biological ones. These processes vary in their effectiveness, cost, and environmental impacts. Among these methods, high-efficiency aproaches such as adsorption [3], oxidation [4], Fenton ozonation [5], and photocatalytic degradation [6,7] are considered promising technologies for treating organic pollutants.

Membranes Technology is widely used to solve crucial energy and environmental problems and is often employed for water purification. For such purification processes, there is a strong need for membranes with the ability to control the transport of small molecules (such as water) or ions. In this regard, polymeric materials have an important role in membrane research and development due to their unique processability, low cost, availability, energy efficiency, simple fabrication at various scales, and minimal use of harmful and toxic chemicals. Polymeric membrane technology has also been used to treat wastewater containing organic pollutants. The integration between the membrane separation process and photocatalysts is a point of great interest.

Cellulose—one of the most abundant biopolymers, generally used as a reinforcing material for fiber technology—is known to be an inexhaustible resource for sustainable natural polymers, in the presence of the growing demand for producing biodegradable and eco-friendly materials [8,9,10,11,12,13]. Moreover, cellulose-based materials have many advantages, such as being environmentally friendly, biocompatible, and cost-effective sources of carbon-based polymers and substrates for developing nanocomposites with novel material properties. One of the most common cellulose derivatives, cellulose acetate (CA), has many unique properties, such as nontoxicity, renewability, cost-effectiveness, and biodegradability. CA is also a widely used polymer for wastewater treatment membranes. The CA membrane usually consists of only one polymeric material and contains an asymmetric structure with a very thin and dense solute used to reject the active layer on a coarse supporting layer [13,14,15,16].

The application of nanocomposite (hybrid) materials as catalysts or adsorbents to remove toxic substances from wastewater has received great attention in the last decade. The adsorbents may be of mineral, organic, or biological origin, such as clay, zeolites, activated carbons, silica beads, polymeric materials, and metal oxides [17,18,19,20,21,22].

Among metal oxides, zinc oxide (ZnO) in its nanostructured form is a favorable semiconducting oxide for application as a nanocomposite polymeric material due to its large variety of low dimensional nanostructures, effective adsorption, high surface reactivity, adsorption capacity, and destructive sorbent ion capacity [14,15,16,17,18,19,20,21,22,23]. ZnO nanostructures (such as nanoparticles, nanowires, nanoflowers, and nanosheets) as multifunctional inorganic nanomaterials have been widely applied in the polymeric membrane modification process [24,25,26,27,28]. However, there are still challenging issues regarding the stability, biocompatibility, and efficiency of these membranes when used in real-life filtration or treatment units. The produced polymer/nanocomposite materials should exhibit superior electrical, mechanical, thermal, and chemical properties; good permeability; thermal stability; microbial resistance; and excellent water selectivity [29,30,31,32,33].

Many studies have been done on nanocomposite membranes in wastewater treatment. For example, Zhao et al. [34] improved the permeability and anti-fouling performance of polyethersulfone ultrafiltration membrane by incorporation of ZnO-DMF dispersion containing nano-ZnO and polyvinylpyrrolidone. The results indicated that the incorporated nano-ZnO was favorable and enhanced compaction resistance and anti-fouling properties by reducing the irreversible fouling resistance of membranes. The inclusion of nano-ZnO also led to thermal stability improvements below 450 °C of the membranes. In another study by Feiya Fu and Lingyan Li [35], cellulose-based ZnO nanocomposite was prepared through one-step coagulation; ZnO nanoparticles were agglomerated into large clusters embedded in the cellulose matrix. The nanocomposite showed good mechanical properties, high thermal stability, excellent UV-blocking properties, and antibacterial activity. Furthermore, Cheng et al. [36] reported a new deacetylated cellulose acetate polydopamine composite nanofiber membrane fabricated by electrospinning and surface modification. The membrane was applied as a highly efficient adsorbent for removing MB from an aqueous solution. The composite nanofibers were employed as highly efficient adsorbents for removing MB from an aqueous solution. In our recent study [37], we reported the fabrication of activated carbon fiber (ACF)/ZnO nanorod (NR) nanocomposite with unique microstructural and photocatalytic properties; the adsorption and photocatalytic activity of the synthesized catalytic adsorbents were compared using MB under UV-light irradiation. The nanocomposite showed excellent photocatalytic activity (~99% degradation of MB in 2 h) compared with bare ZnO-NR and ACF. Additionally, a recycling experiment demonstrated the stability of the photocatalyst. The enhanced photocatalytic activity may be related to the synergetic adsorption–photocatalytic degradation effect of ACF and ZnO nanostructures.

The present study describes the development of a new polymeric membrane based on CA-ZnO nanocomposite. ZnO nanostructures were impregnated or grown in the cellulose acetate polymer matrix using simple and cost-effective sonication and hydrothermal processes in this nanocomposite. The pristine CA, CA-ZnO-NP, and CA-ZnO-NW membranes were prepared and characterized, and their photocatalytic activity toward MB in aqueous solutions were investigated using a UV lamp and a sunlight simulator. The membranes were characterized using XRD, SEM, FT-IR, TGA, UV-vis, and contact angle tests. A membrane separation process coupled with photocatalysts can act as a barrier for MB dye to enhance the photocatalytic degradation and treatment process of wastewater.

## 2. Materials and Methods

### 2.1. Materials

Zinc nitrate, zinc acetate, ammonium carbonate, NaOH, and ethanol obtained from Sigma Aldrich Chemical Co, St. Louis, MO, USA, were used to prepare ZnO nanoparticles and ZnO nanowires. Hexamethylenetetramine (HMTA) and cellulose acetate and dimethylformamide (DMF) were obtained from Sigma Aldrich Chemical Co, USA, to prepare the membrane casting solution. 

### 2.2. Synthesis of ZnO Nanoparticles

ZnO nanoparticles were prepared by hydrothermal method. An equimolar solution of 0.5 M of zinc nitrate Zn(NO_3_)_2_ and ammonium carbonate (NH_4_)_2_CO_3_ was dissolved and stirred separately in deionized water at room temperature. The precursor was prepared by adding Zn(NO_3_)_2_ solution dropwise into the vigorously stirred (NH_4_)_2_CO_3_ with a molar ratio of 1:1.5, forming white precipitates. These precipitates were collected by filtration and repeatedly rinsed with deionized water. The rinsed precipitates were dried at 90 °C for 6 h to form the final ZnO-NPs. 

### 2.3. Synthesis of ZnO Nanowires

#### 2.3.1. Preparation of ZnO Seed Solution

To obtain ZnO nanoparticle seeds, two solutions were prepared: 1 mM of zinc acetate in 100 mL ethanol and 1 mM of NaOH in 20 mL ethanol, which were stirred with heating at 50 °C. NaOH solution was added to the zinc acetate solution dropwise while stirring. Then, the mixed solutions were heated in the water bath for 1 h at 60 °C.

#### 2.3.2. Growth Solution Preparation

For the growth of ZnO nanoparticles, 1 mM Hexamethylenetetramine (HMTA) in 100 mL ethanol and zinc nitrate in 100 mL ethanol were prepared and mixed with stirring.

#### 2.3.3. Growth of ZnO Nanowires

A few drops of the seed solution were added onto the glass substrate by spin coater to make a layer, and then it was dried on a hot plate at 40 °C. This step was repeated three times. Then, the substrate was placed in the growth solution with the seed layer facing the bottom to form the nanowires. The growth solution with the substrate was placed in a water bath at 90 °C for 2 h. After that, the substrate was dried at room temperature. ZnO nanowires were scraped (white layer) from the top of the substrate.

### 2.4. Membrane Preparation

To obtain the optimum ZnO concentrations in the CA matrix, several experiments were done for ZnO content (0.1%, 0.5%, 1%, and 5% wt%). The best membrane performance obtained was with ZnO (5%). A control cellulose acetate polymeric membrane was prepared using the phase inversion method, in which 15% weight cellulose acetate was dissolved in 85% dimethylformamide (DMF) under constant mechanical stirring for 1–2 h at 40 °C. The obtained homogeneous solution was allowed to stand in the air to remove the air bubbles. The cellulose acetate impregnated with ZnO nanoparticles was prepared similarly, except that 5% ZnO nanoparticles were added to the membrane solution.

All membranes were prepared by casting using a spin coater at a speed of 1000 rpm for 10 s using the model “Laurell WS- 650-23”, Laurell Technologies Corporation, North Wales, PA, USA.

### 2.5. Membrane Casting

**Figure 1:**
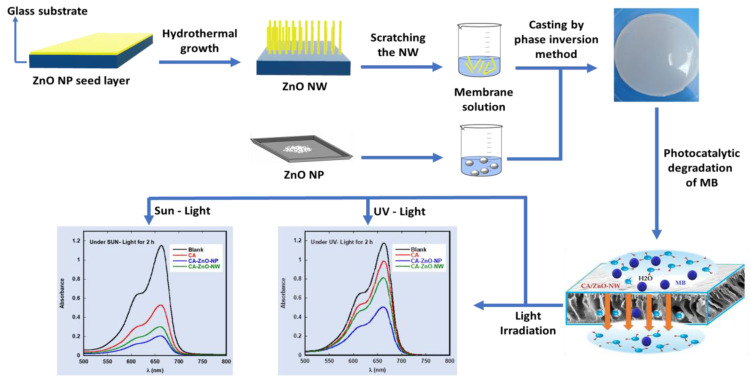
Schematic illustration of CA-ZnO-nanostructured membrane preparation and testing processes.

CA membrane casting using the CA\DMF solution on a mirror was performed using spin-coating techniques. After casting, the mirror was immersed in a deionized water bath for 3–4 min until the membrane separated from the mirror. An optical sample image of the prepared membranes is shown in Figure 1.

### 2.6. Photocatalytic Activity Evaluation

The photocatalytic activity of all prepared CA-ZnO nanocomposites with various ZnO nanostructures was evaluated with respect to MB photocatalytic degradation. Then, the nanocomposite with the best photocatalytic activity and performance was subjected to a detailed study. The photocatalytic activity of the prepared photocatalysts (pristine CA, CA-ZnO-NP, CA-ZnO-NW) were evaluated by observing the degradation of (MB) pollutants. MB stock solution was prepared by dissolving it in distilled water at 100 mg L^−1^ into a 250 mL beaker. Then, 10 mg of CA, CA-ZnO-NP, and CA-ZnO-NW photocatalysts (each in a separate 10 mL bottle) were added to the MB solution at a pH of 7. To ensure adsorption/desorption equilibrium of MB on these photocatalysts, the solutions were mixed using a magnetic stirrer in dark medium for 30 min and centrifuged at 3000 rpm for 5 min. The UV-vis absorption spectrum of the solution was measured in the range of 200–800 nm. Then, the mixture was exposed to a UV lamp (6 Watts, λ = 365 nm). The initial MB concentration was 50 mg L^−1^ and the temperature of the reaction solution was maintained at 30.0 ± 0.5 °C. Ten samples were taken with fixed time intervals of 10 and 20 min after UV radiation, and their absorption spectrum was recorded. The residual concentration of MB dye in the solution was measured to have a major peak at 664 nm using a UV-visible spectrophotometer.

The same procedure was applied for sunlight irradiation using a sunlight simulator (Peccell’s sunlight simulator, PEC-L15, Peccell Technologies, Inc., Yokohama, Japan) with a sunlight irradiance of 100 mW/cm^2^. 

A blank sample (containing no dye) was used as a control, and a calibration curve of absorbance versus concentration was constructed. Samples were collected at 15 min time intervals (up to 2 h) and centrifuged to remove the catalyst before analysis. The degradation efficiency of the MB was calculated by Equation (1).

% Degradation = (C_0_ − C_t_) × 100/C_0_ (1)

where C_0_  =  initial MB concentration and Ct  =  MB concentration after time t.

To detect MB degradation products in the presence of CA-ZnO-NP membranes, total organic carbon (TOC), ammonium, nitrate, and sulfate concentrations were determined in the presence and absence of CA-ZnO-NP membranes under UV-light and sunlight irradiation using Lovibond kits and methods: TOC-1K and TOC-2K method 381, AMMONIA No.1 and AMMONIA No.2 method 60, VARIO Nitrate Chromotropic method 265, and Vario sulfa 4 method 355, respectively. All tests were analyzed using a spectrophotometer (Lovibond SpectroDirect, Dortmund, Germany) at 330–900 nm. Zn concentrations for leaching tests were analyzed quantitatively using atomic absorption flame emission spectroscopy ( Shimadzu (AA-6200), Shimadzu Co., Kyoto, Japan).

## 3. Results

### 3.1. X-ray Diffraction (XRD)

**Figure 2:**
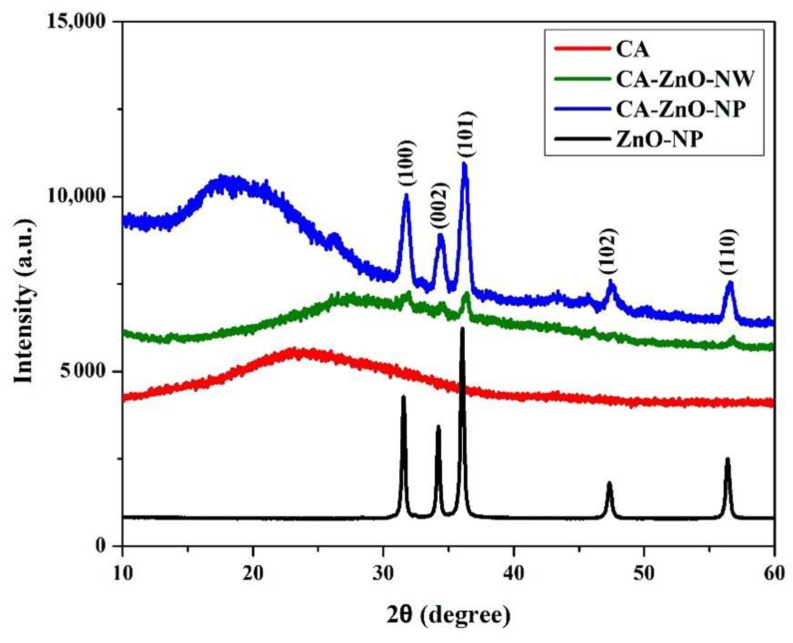
XRD patterns of the pristine CA, CA-ZnO-NP, and CA-ZnO-NW membranes.

X-ray diffraction spectra for the samples have been obtained using Cu-Kα radiation (λ = 1.542 Å). Figure 2 shows the X-ray diffraction spectra for ZnO-NP, ZnO-NW, CA, CA-ZnO-NP, and CA-ZnO-NW. The CA-ZnO-NP membrane, CA-ZnO-NW membrane, and ZnO-NP samples displayed typical major diffraction peaks at 2θ = 31.60°, 34.24°, 36.17°, 47.38°, and 56.32°, which were ascribed to the (100), (002), (101), (102), and (110) planes of the ZnO crystal structure form, respectively [38]. The CA membrane, CA-ZnO-NP membrane, and CA-ZnO-NW membrane showed broad peaks at 2θ = 23.30°, 27.23°, 18.17°, respectively, confirming the presence of cellulose acetate matrix [39].

The growth of nanowires was in random orientations, which may be the reason for the presence of peaks with relatively low intensities and crystallinity for the CA-ZnO-NW nanostructure compared to those for the CA-ZnO-NP ones. However, samples with CA content exhibited an amorphous nature in the Brag’s angle (2θ) range between 10° and 30°.

An XRD analysis of the spent photocatalysts was performed after 3 days of photocatalytic experimentation. The preliminary results of the XRD pattern comparison between the pristine and the spent catalyst revealed that the patterns were remarkably similar. No changes in either XRD peak intensities or in the inter-planar spacing were observed after 3 days of the photocatalytic experimentation. This indicates that the spent photocatalysts had chemical and morphological stability.

**Figure 3:**
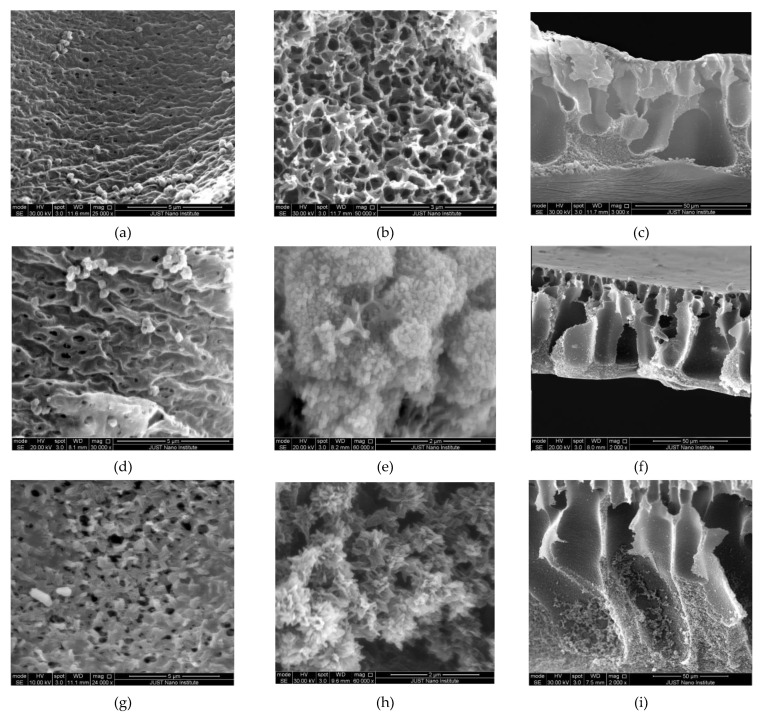
SEM morphologies of the pristine CA membrane (**a**–**c**), the CA-ZnO-NP membrane (**d**–**f**), and the CA-ZnO-NW membrane (**g**–**i**). Images on the left, middle, and right correspond to the top surface, bottom surface, and cross-sectional images of the membranes, respectively.

Figure 3 shows the SEM morphologies of the pristine CA, CA-ZnO-NP, and CA-ZnO-NW membranes. In a typical membrane, the water and ion permeability is determined by the active layer on the top surface, while the structural parameters influence the microstructure of the porous support layer. In phase inversion membranes, void microstructures are usually divided into two cases: a sponge-like structure and finger-like macro-voids. The pristine CA membrane (Figure 3a) showed a uniform and dense top surface with a few randomly scattered grains, which may be attributed to some impurities present during the fabrication process. However, the top surface of the CA-ZnO-NP and CA-ZnO-NW (Figure 3d,g) were less dense, and the assembled nanostructures (nanoparticles and nanowires) were well distributed and dispersed over the whole surfaces and impregnated with the CA-polymer matrix. Moreover, a few pores of various sizes (less than 1 μm) could be easily seen in all membranes. Micrographs of the membranes’ bottom layers are presented in Figure 3b,e,h. The bottom layer of the pristine CA membrane demonstrated a highly porous layer with a well ordered micro-network. However, the ZnO-NP and ZnO-NW nanostructures were incorporated in the bottom surface of the membranes (Figure 3e,h) as an agglomeration of ZnO nanostructures with various sizes and shapes (nanowires, nanoparticles, nanoflowers). These incorporated particles in the active layer improve water permeability by providing alternative flow paths to water molecules. In this regard, choosing the optimum concentrations of the ZnO nanostructures is crucial in membrane fabrication, since larger nanostructure agglomerations can cause a structural defect in the membrane. The ZnO nanostructures had obvious clustering/agglomeration visible on the top layer and in the cross section of membranes (Figure 3e,f,h,i). However, serious agglomeration for higher concentrations may lead to pore plugging, which may negatively affect the membrane performance. For the pristine CA membrane, cross-sectional SEM images (Figure 3c,f,i) showed that a sponge-like structure occupied most of the support layer, and only a few finger-like macro-voids were present. However, longer vertical fingers with numerous shapes were formed in the CA-ZnO nanocomposite membrane support layers. The growth of the ZnO nanostructures can easily be seen in the cross-sectional images of the CA-ZnO-NP and CA-ZnO-NW membranes (Figure 3f,i).

### 3.2. Fourier-Transform Infrared Spectroscopy (FTIR)

**Figure 4:**
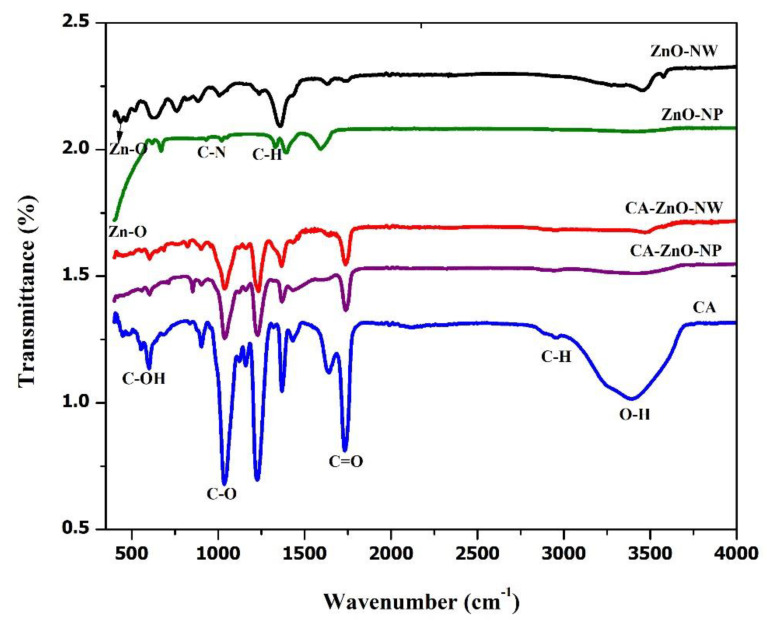
FTIR spectra of the pristine CA membrane, the CA-ZnO-NP membrane, and the CA-ZnO-NW membrane.

Figure 4 shows the FTIR spectra for the ZnO-NP, ZnO-NW, CA, CA-ZnO-NP, and CA-ZnO-NW membranes. The infrared absorption spectra of the samples were observed in the 400–4000 cm^−1^ wavenumber range. For all membranes, the broad peaks at 607 cm^−1^ were assigned to the aromatic C-OH bond polymer chain. The peak of C-O stretching was present at 1029 cm^−1^. C=O exhibited a strong asymmetric mode of vibration at 1733 cm^−1^. A small peak at 2958 cm^−1^ is due to the stretching of the C-O group of cellulose. Moreover, the peak at 3396 cm^−1^ endorses the O-H bond of the membranes. For the ZnO-NP and ZnO-NW samples, the presence of absorption bands at 417 and 461 cm^−1^ are due to Zn-O bond stretching in the ZnO structure. The C=O peak is at 1593 cm^−1^ in the ZnO-NP sample and 1630 cm^−1^ in the ZnO-NW sample. In the CA-ZnO-NP and CA-ZnO-NW samples, the Zn-O peaks were present at 428 cm^−1^ and 401 cm^−1^, respectively.

### 3.3. Thermogravimetric Analysis (TGA)

**Figure 5:**
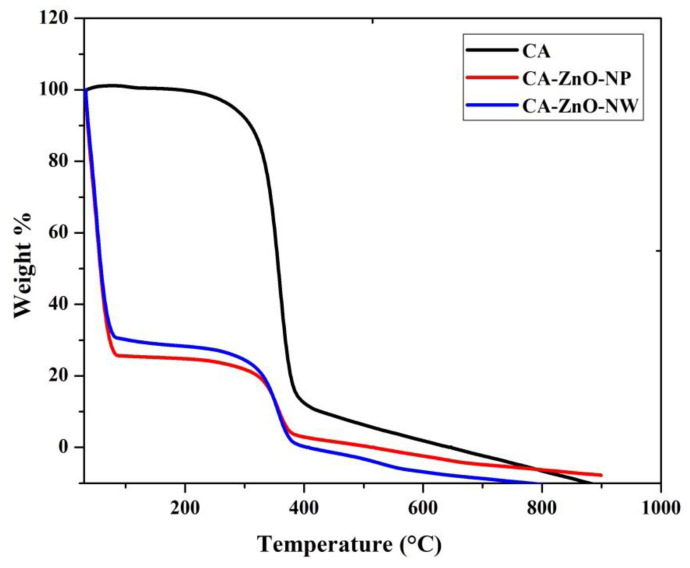
TGA data of the pristine CA membrane, the CA-ZnO-NP membrane, and the CA-ZnO-NW membrane.

TGA curves of CA, CA-ZnO-NP, and CA-ZnO-NW membranes are illustrated in Figure 5. Generally, there were three steps of degradation in the TGA curve for all membranes. The first step started from 52 °C to 201 °C for the CA membrane and from 33 °C to 73 °C with 74.34% mass change for the CA-ZnO-NP membrane and 69.63% mass change for the CA-ZnO-NW membrane. This weight loss for prepared membranes can be ascribed to evaporation of remaining solvents, the evaporation of residual absorbed water, and easily volatile products of membrane thermal degradation. In the second step, there was a sharp weight drop from 287 °C to 400 °C with 88.76% mass change for the CA membrane, from 302 °C to 397 °C with a mass change of 19.30% for the CA-ZnO-NP membrane, and from 302 °C to 387 °C with a mass change of 25.35% for the CA-ZnO-NW membrane. The second step represents the main thermal degradation of the cellulose acetate chains. The last step for all membranes started at 433 °C, 410 °C, and 402 °C for the CA, CA-ZnO-NP, and CA-ZnO-NW membranes, respectively, indicating the carbonization of the degraded products to ash. These three steps may correspond to steps suggested by [40].

### 3.4. Water Contact Angle Measurement 

The contact angle tests for the membranes were conducted using an experimental setup developed at our lab. An apparatus that is quite efficient, precise, and low cost was used for static contact angle measurements, which proved to be adequate for measuring contact angles of a ~10 µL DI water droplet injected from an automated syringe dispenser. The droplet image was taken using “Stereomicroscopes SZO-8, Optika, Ponteranica (BG), Italy” Stereo zoom microscope equipped with a digital camera. The profile of the drop was processed with ImageJ free source software (V 1.8.0_172). The drop-shape analysis was based on a sessile drop technique by placing a water drop on a dry membrane surface exposed to the surrounding air and measuring the contact angle that the drop makes with the membrane surface. Since the static water contact angle may change with time for various reasons, a snapshot picture of the drop with the membrane surface was taken during the first few seconds after dropping, through which the contact angle was almost constant. The ratio of the cohesive forces to adhesive forces between the water droplet and the membrane surface provides an indication of the size of the contact angle. Reported contact angle data was obtained by averaging three points on each surface. For each membrane, two different surfaces were tested.

**Figure 6:**
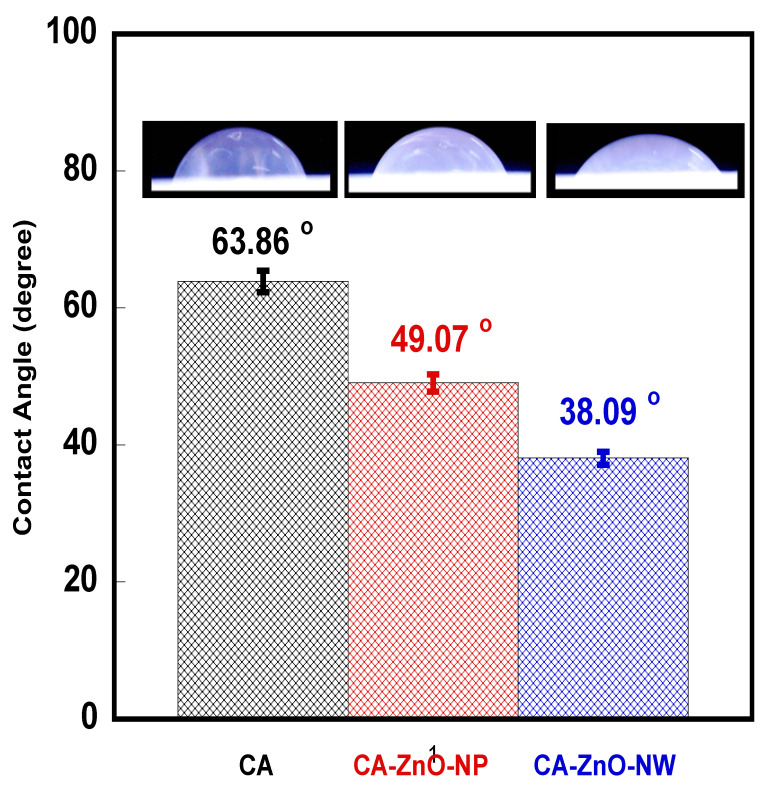
Contact angle measurements of the pristine CA membrane, the CA-ZnO-NP membrane, and the CA-ZnO-NW membrane. The insets show a photograph of a water droplet on each membrane.

Figure 6 shows the contact angle measurement of the pristine CA membrane, the CA-ZnO-NP membrane and the CA-ZnO-NW membrane. The insets show a photograph of a water droplet on each membrane. A gradual decrease in the contact angle was observed for CA-ZnO-NP (49.07° ± 1.22°) and CA-ZnO-NW (38.09° ± 0.95°), compared to pristine CA (63.86° ± 1.60°). A consistent suppression in the contact angle with the addition of ZnO-NP and ZnO-NW proved that the addition of hydrophilic ZnO nanostructures on the surface of the CA membrane improves the hydrophilicity of the membrane. 

The results indicate hydrophilic surfaces for all membranes. The decrease in the contact angle (improvement of the hydrophilicity of the CA membranes) with the introduction of ZnO nanostructures into the CA matrix could have arisen due to the crystallinity and microstructural changes that occur during the growth of these nanostructures inside and on the boundaries of the CA porous network. Moreover, the surface of ZnO can be hydrophobic or hydrophilic depending on the ZnO/water interfacial interactions.

### 3.5. Photocatalytic Activity

**Figure 7:**
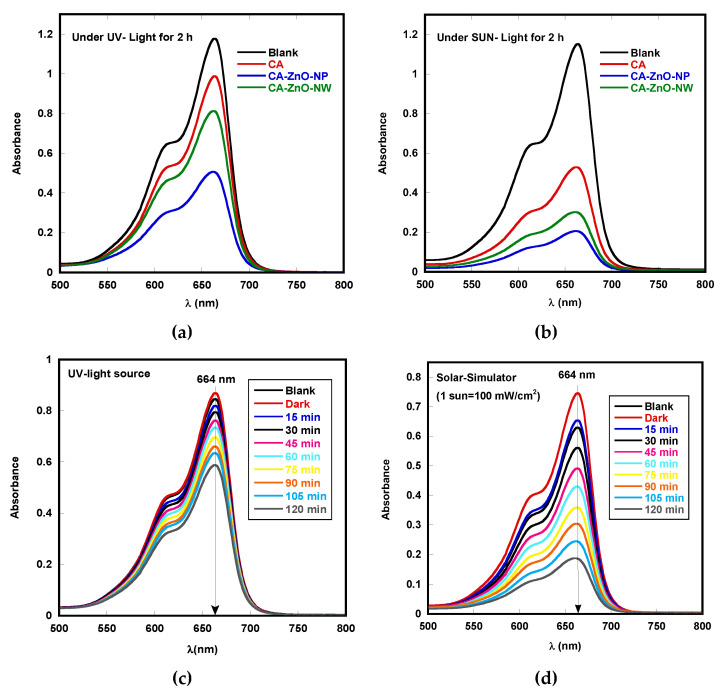
Photocatalytic degradation of MB by pristine CA, CA-ZnO-NP, and CA-ZnO-NW membranes, each after (**a**) UV-light irradiaton and (**b**) sunlight irradiation for 2 h; MB degradation in the CA-ZnO-NP membrane under (**c**) UV-light irradiation and (**d**) sunlight irradiation every 15 min.

Figure 7 presents the photocatalytic degradation of MB by the pristine CA, CA-ZnO-NP, and CA-ZnO-NW membranes after UV-light and sunlight irradiation. It can be observed in all samples that the absorbance intensity of the MB absorption peak (664 nm) decreased gradually for both UV-light and sunlight irradiation. The maximum degradation of MB under UV-light was observed in the CA-ZnO-NP membrane (about 60%) after 2 h compared to the blank sample (Figure 7a). However, a more pronounced decrease (of more than 80%) in the MB major absorption peak intensity was seen using the CA-ZnO-NP membrane in sunlight (Figure 7b). This suggests that CA-ZnO-NP membranes are a proper candidate for the photocatalytic degradation of MB compared to other membranes. Moreover, to optimize the experimental conditions for the best photocatalytic performance of the membranes, a systematic study was performed to find the optimum ZnO-NW, ZnO-NP, and MB concentrations for samples with excellent performance.

Based on the obtained preliminary result, a systematical and detailed study was conducted for the CA-ZnO-NP membrane photocatalytic activity toward MB dye under both UV-light and sunlight irradiation. 

The presented results revealed that the optimum results were obtained with samples of 20% weight. CA-ZnO-NP membranes were loaded in 5 mg/L MB solution (pH ≈ 7) for 120 min. The photocatalytic activity of the optimized samples was evaluated by observing the degradation of MB in the presence of the CA-ZnO-NP membrane under UV-light irradiation (Figure 7c).

During the first 30 min (dark adsorption test), all samples showed a stable adsorption–desorption equilibrium in MB solution. For comparison, we also studied the photocatalytic degradation of MB by direct photolysis, i.e., without using a catalyst under identical experimental conditions. A negligible degradation of MB (~1%) over 2 h was observed, indicating that the properties of methylene blue are more stable. CA-ZnO-NP showed ~30% of the MB dye reduction during the first 2 h of UV-light irradiation. The photocatalytic results under sunlight irradiation in the presence of CA-ZnO-NP showed a more rapid degradation of MB (about 75%) (Figure 7d) compared to results obtained under UV-light irradiation. The results show that CA-ZnO-NP possesses the photocatalytic ability to degrade MB at different levels under both UV-light and sunlight irradiation.

The degradation of MB in the presence of CA-ZnO-NP membranes under UV-light and sunlight irradiation for an irradiation period of 120 min leads to the conversion of nitrogen and sulfur atoms in MB molecules into inorganic ions and gasses. As reported in Table 1, the reduction in TOC and the increase in ammonium (NH_4_^+^), nitrate (NO_3_^−1^), and sulfate (SO_4_^−2^) concentrations confirmed the mineralization and decomposition of MB [41] and agree well with our degradation findings. 

**Figure 8:**
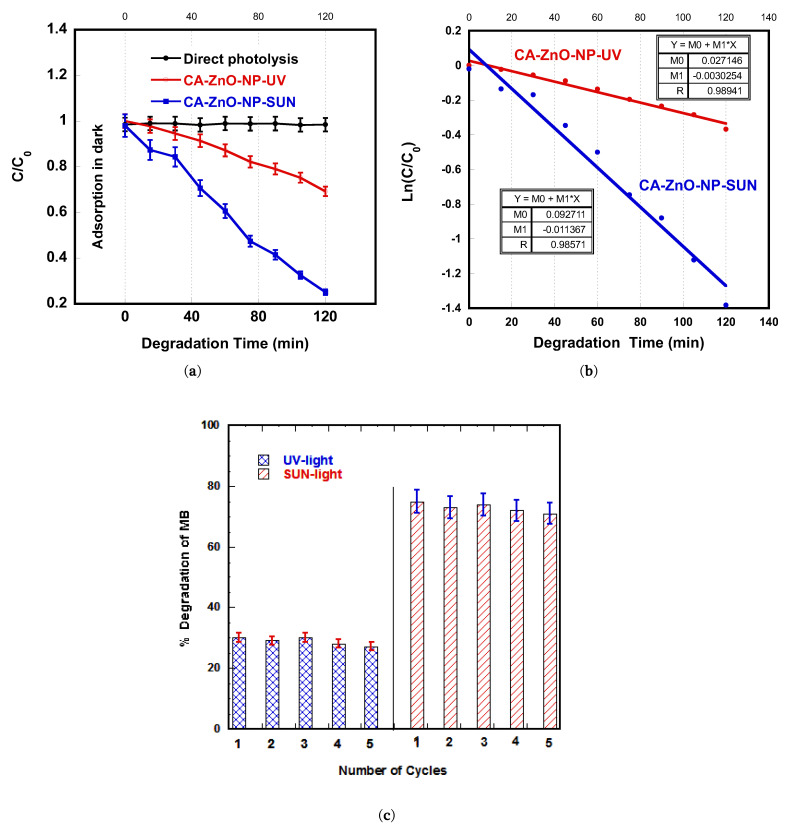
(**a**) Plots of C/C_0_ vs. irradiation time for photocatalytic degradation of MB in the presence of CA-ZnO-NP membranes under UV-light and sunlight irradiation. (**b**) Plots of ln(C/C_0_) vs. irradiation time for photocatalytic degradation of MB in the presence of CA-ZnO-NP membranes under UV-light and sunlight irradiation. (**c**) Cyclic runs showing the photocatalytic degradation of MB in the presence of CA-ZnO-NP membranes under UV-light and sunlight irradiation.

To gain a deep insight into the kinetic mechanism during the photocatalytic process, the C/C_0_ versus degradation time plots are illustrated in Figure 8a. The results indicate that the photodegradation process proceeded gradually with irradiation time, with a much steeper decrease for the CA-ZnO-NP membrane under sunlight compared to that for UV-light irradiation. The photodegradation of an organic dye generally occurs on the photocatalyst surface; the surface area becomes a significant issue in the photocatalysis process. The results clearly indicate that MB rapidly degraded in the presence of CA-ZnO-NP under sunlight irradiation, compared to under UV-light irradiation.

For better illustration of the degradation rates, the rate constants (k) for the degradation of MB were calculated from the well known kinetic equation ln(C/C_0_) = k·t and plotted in Figure 8b, where the degradation rate constant (k) was calculated from the slope of the line. For UV-light and sunlight irradiation, the rate constants were calculated to be 0.0030 and 0.0114 min^−1^, respectively. The CA-ZnO-NP membrane exhibited higher photocatalytic activity under sunlight (more than three times) than that under UV light. The linear plots illustrated in Figure 8b reveal that the degradation process of MB followed first order kinetics, which is in full agreement with [37]—A recently published study on ACF-ZnO-NR nanocomposites. This may be attributed to the high surface area contributed by CA fibers; high ZnO surface ratio; small ZnO-NP size; and the wide spectrum of the sunlight, which increases the number of active sites on the membrane surface and thus leads to higher reactivity [42].

CA-ZnO-based nanocomposite photocatalytic activity, which may be attributed to the strong electronic interaction between CA and ZnO, has been reported by Abdalkarim et al. [43]. Further findings from previous studies that investigated different polymeric ZnO nanocomposites with respect to MB degradation are summarized in Table 2. 

Additionally, a photocatalytic mechanism for CA-ZnO-NP nanocomposite has been also reported. According to Huang et al. [44], during UV-light and sunlight irradiation, the photogenerated electron-hole pairs due to the ZnO-NP light absorption migrate to the surface of ZnO and react with O_2_ and H_2_O absorbed on the surface of ZnO to generate O_2_^−^ and OH^−^, which may contribute towards MB degradation through a direct oxidation process. We believe that, although CA-ZnO-NP nanocomposite possesses dual absorption of UV radiation and visible light from sunlight for enhanced photocatalytic activity, the generation of reactive radical species in each electromagnetic radiation is quite different and requires further systematic investigation. Therefore, this may also lead to the application of sunlight radiation alone in the wastewater treatment industry, since sunlight is a readily available type of light with both UV and visible light components. 

The reproducibility of the CA-ZnO-NP membrane with respect to the photodegradation of MB under UV-light and sunlight irradiation is shown in Figure 8c. The photodegradation was monitored for five cycles, each for 120 min. After each cycle, the membrane was taken off from the MB solution, washed thoroughly with water, and then transferred to a fresh MB solution. The figure shows no significant change in the removal efficiency or photocatalytic degradation during the five consecutive cycles under UV-light and sunlight irradiation.

In addition, Zn ion leaching from the membranes was evaluated at the end of each cycle after the photocatalytic process; the concentration for each membrane was determined quantitatively using Shimadzu (AA-6200) atomic absorption flame emission spectroscopy. The results showed a total Zn leaching of only 0.38% (UV-light irradiation) and 0.36% (sunlight irradiation) of total membrane Zn content after five cycles of operation. This indicates the excellent durability, recyclability, and reproducibility of the CA-ZnO-NP membranes with respect to the photodegradation of MB.

## 4. Conclusions

In this work, the polymeric CA-ZnO nanocomposite membranes were easily fabricated using the phase inversion method and sono-hydrothermal synthesis technique. ZnO nanostructures (nanoparticles and nanowires) were applied to fabricate a CA-based composite membrane to enhance their performance by investigating the MB photocatalytic degradation in aqueous solutions. Compared to pristine the CA membrane, the CA-ZnO-NP and CA-ZnO-NW membranes exhibited enhanced photocatalytic activity and degradation of MB. The results obtained via XRD and SEM demonstrate a clear modification of their structural and morphological properties due to impregnation of ZnO nanostructures. The optimum concentrations, size, and shape of the impregnated ZnO nanostructures (nanoparticles, nanowires, nanoflowers, etc.) are crucial for membrane performance, since the presence of large nanoclusters and agglomerations may create structural defects which may have a negative impact on the membrane quality and performance. Moreover, the photocatalyst could also be conveniently recovered and used for subsequent runs at sustained proficiencies. Besides the degradation of organic dye compounds, the inhibition of microbial growth by the ZnO nanostructures parallel to UV-light and sunlight irradiation could be potential topics for future investigation.

## Figures and Tables

**Table 1:** The degradation products of MB in the presence of CA-ZnO-NP membranes under UV-light and sunlight irradiation for an irradiation period of 120 min.

Parameter	Reading before Degradation	Reading after Degradation with CA/ZnO under UV-Light Irradiation	Reading after Degradation with CA/ZnO under Sunlight Irradiation
TOC (mg/L)	2.1	0.76	0.43
Ammonium (mg/L)	0	0.4	0.6
Nitrate (mg/L)	0	1.1	4.1
Sulfate (mg/L)	0	7.8	9

**Table 2:** Comparison of MB Dye degradation in different studies.

Photo-Catalyst Composition	MB Concentration	Irradiation Time (min)	Rate Constant min^−1^	% Degradation	Type of Irradiation	Reference
Polyaniline/Zinc oxide						[45]
1:1	50 (mg/L)	120	0.006	52.0	Visible light	
1:2	50 (mg/L)	120	0.01944	90		
1:3	50 (mg/L)	120	0.00724	58.9		
Polyaniline/Zinc oxide						[46]
PANI/0.5 wt% ZnO	10 (mg/L)	180	NS	76	Visible light	
Polyaniline/Zinc oxide	1 × 10^−5^ M	300	0.011	79	UV light	[47]
(catalyst concentration: 0.4 mg/mL)	1 × 10^−5^ M	300	0.02405	97	Sunlight	
Polyimide/ZnO						[48]
0.2 M ZnO	5 (mg/L)	150	0.0166	92	UV light	
0.5 M ZnO	5 (mg/L)	150	0.0283	98		
1.0 M ZnO	5 (mg/L)	150	0.0216	96		
2.0 M ZnO	5 (mg/L)	150	0.010	75		
Poly (methyl methacrylate) (PMMA)/ZnO						[29]
Powders		240	0.041	60	UV light	
composite film		240	0.026	40		
flat film		240	0.017	30		
Cellulose/ZnO 35 mg of nanopowders	3.25 (g/L)	300	0.1174 h^−1^	79	UV light	[49]
Cellulose acetate/ZnO		120	0.0030	30	UV light	This work
Cellulose acetate/ZnO		120	0.0114	75	Sunlight	

NS: not studied.

## Data Availability

The data presented in this study are available on request from the corresponding author.
